# Online interventions to address HIV and other sexually transmitted and blood‐borne infections among young gay, bisexual and other men who have sex with men: a systematic review

**DOI:** 10.1002/jia2.25017

**Published:** 2017-11-01

**Authors:** Rod Knight, Mohammad Karamouzian, Travis Salway, Mark Gilbert, Jean Shoveller

**Affiliations:** ^1^ British Columbia Centre on Substance Use Vancouver Canada; ^2^ School of Population and Public Health University of British Columbia Vancouver Canada; ^3^ HIV/STI Surveillance Research Center, and WHO Collaborating Center for HIV Surveillance Institute for Futures Studies in Health Kerman University of Medical Sciences Kerman Iran; ^4^ British Columbia Centre for Disease Control Vancouver Canada

**Keywords:** HIV, sexually transmitted and other blood‐borne infections, online, Internet, intervention, web‐based, gay, bisexual, men who have sex with men, young men

## Abstract

**Introduction:**

Globally, young gay, bisexual and other men who have sex with men (gbMSM) continue to experience disproportionately high rates of HIV and other sexually transmitted and blood‐borne infections (STBBIs). As such, there are strong public health imperatives to evaluate innovative prevention, treatment and care interventions, including online interventions. This study reviewed and assessed the status of published research (e.g. effectiveness; acceptability; differential effects across subgroups) involving online interventions that address HIV/STBBIs among young gbMSM.

**Methods:**

We searched Medline, Embase, PsycINFO, CINAHL, and Google Scholar to identify relevant English‐language publications from inception to November 2016. Studies that assessed an online intervention regarding the prevention, care, or treatment of HIV/STBBIs were included. Studies with <50% gbMSM or with a mean age ≥30 years were excluded.

**Results:**

Of the 3465 articles screened, 17 studies met inclusion criteria. Sixteen studies assessed interventions at the “proof‐of‐concept” phase, while one study assessed an intervention in the dissemination phase. All of the studies focused on behavioural or knowledge outcomes at the individual level (e.g. condom use, testing behaviour), and all but one reported a statistically significant effect on ≥1 primary outcomes. Twelve studies described theory‐based interventions. Twelve were conducted in the United States, with study samples focusing mainly on White, African‐American and/or Latino populations; the remaining were conducted in Hong Kong, Peru, China, and Thailand. Thirteen studies included gay and bisexual men; four studies did not assess sexual identity. Two studies reported including both HIV+ and HIV− participants, and all but one study included one or more measure of socio‐economic status. Few studies reported on the differential intervention effects by socio‐economic status, sexual identity, race or serostatus.

**Conclusion:**

While online interventions show promise at addressing HIV/STBBI among young gbMSM, to date, little emphasis has been placed on assessing: (i) potential differential effects of interventions across subgroups of young gbMSM; (ii) effectiveness studies of interventions in the dissemination phase; and (iii) on some “key” populations of young gbMSM (e.g. those who are: transgender, from low‐income settings and/or HIV positive). Future research that unpacks the potentially distinctive experiences of particular subgroups with “real world” interventions is needed.

## 1 Introduction

Globally, young men's engagement with HIV/sexually transmitted and blood‐borne infections (HIV/STBBI) care remains low, particularly in marginalized subgroups such as young gay, bisexual and other men who have sex with men (gbMSM) 1, 2, 3. For example, gbMSM experience high numbers of new HIV diagnoses across low‐, medium‐ and high‐income settings, with a notable increase in new HIV diagnoses among young adult and adolescent gbMSM over the past decade 4, 5. As such, HIV has been described as a “re‐emerging epidemic” among younger generations of gbMSM 1, particularly in vulnerable subgroups of young gbMSM (e.g. those who are: economically deprived; ethno‐racial minorities; living in regions with discriminatory policies and/or political and cultural influences) 6. At the same time, the increasing incidence among young gbMSM of viral (e.g. syphilis, Hepatitis C) and bacterial infections (e.g. gonorrhoea) 7, 8 further signals the importance of developing new and innovative intervention approaches to meet the needs of today's generation of young gbMSM. As such, there are strong public health imperatives to identify effective prevention, treatment and care interventions that address HIV and other STBBIs among young gbMSM 2.

The Internet provides a medium to address the prevention, care, and/or treatment of HIV/STBBIs 9, 10, 11, particularly among youth^1^ less than 30 years of age – a highly “connected” generation 12, including among young gbMSM 13, 14. As such, many new online health promotion interventions have emerged during key transitional periods in the life course of the current generation of young gbMSM (e.g. as they move from childhood into adolescence or early adulthood). Previous research has illustrated how online interventions can change both mediators of safer sex (e.g. knowledge about sexual health, self‐efficacy), in addition to behavioural (e.g. condom use, testing) and biomedical outcomes (e.g. incident infections) 15, 16. Online approaches to intervention are also considered scalable and cost‐effective and may provide opportunities to overcome challenges with delivering HIV/STBBI interventions to “hidden” or “hard‐to‐reach” populations who may not otherwise access in‐person programmes 9, including young gbMSM 17. Moreover, global access to the Internet via a variety of devices (e.g. mobile phones, smartphones, notebooks, desktop computers, and tablets) is widespread, particularly among youth <30 years, including within many low‐, middle‐ and high‐income settings 18, 19, 20. Policy makers and intervention strategists are also increasingly aware that the Internet provides opportunities to meet young people “where they are at,” including via social and sexual networking applications (“apps”) which are often widely used by young gbMSM. For instance, mobile apps like *Grindr*,* Scruff*, and *Tinder* have millions of gbMSM users active across most areas of the globe 21, with a recent systematic review identifying that the majority of gbMSM using geosocial networking apps are ≤30 years of age 13. As such, while web‐based technologies may facilitate sexual risk behaviour among young gbMSM (e.g. “low‐threshold” access to multiple and concurrent partners), they also provide innovative and promising opportunities to provide the right intervention to the right groups of gbMSM at the right time 22.

While online approaches have shown promise in providing sexual health promotion and care to young people, less is known about how online interventions can address the prevention, care, and/or treatment of HIV/STBBIs among young gbMSM, and reviews of online approaches to address HIV/STBBIs have been notably absent 23. This article provides a comprehensive review of the literature of online interventions that aim to address HIV and other STBBIs among young gbMSM by answering two primary research questions: (i) *What is the status of research (e.g. effectiveness; acceptability) involving online interventions to address HIV/STBBIs among young gbMSM?*; and (ii) *What are the differential intervention effects according to intervention type (e.g. behavioural, biomedical, structural), social positioning (e.g. by SES; sexual identity) and research design?* By answering these research questions through a systematic review of the peer‐reviewed literature, our aim is to identify effective intervention strategies and to inform a renewed research agenda regarding the development of evidence‐based online interventions for young gbMSM.

## 2 Methods

The research questions, outcome measures, search strategy, study selection process, and data analysis plan were based on an internal unpublished protocol developed prior to the initiation of the activities involved in this review process.

### 2.1 Search strategy

Following the Systematic Reviews and Meta‐Analyses (PRISMA) checklist 24 (see Appendix S1), we searched for studies related to online STBBI/HIV prevention and care among young gbMSM that were in English and published in a peer‐reviewed journal in the following databases from inception through 15 November 2016: *Medline*,* PsycINFO*,* CINAHL*,* EMBASE*, and *Google Scholar* (the first 300 hits) 25. Search terms were combined using appropriate Boolean operators and included subject heading terms or key words for four key themes and were tailored to fit each database requirements: men who have sex with men (e.g. homosexuality OR bisexuality OR men who have sex with men OR gay men OR MSM) AND HIV/STI (e.g. HIV OR AIDS OR STI/STD OR gonorrhea OR syphilis OR chlamydia OR herpes OR hepatitis) AND intervention (e.g. prevention OR intervention OR programme OR implementation OR evaluation) AND online (e.g. Internet‐based OR web‐based OR online OR e‐health). Hand searches of the bibliographies of relevant published works and previous reviews were also performed. Our full electronic search strategy is included as a supplemental file.

### 2.2 Eligibility criteria

The population, interventions, comparisons, outcomes and study designs considered for review are listed in Table 1. Studies were only included if they had provided post‐intervention results.

**Table 1:** Population, interventions, comparisons, outcomes and study design (PICOS) criteria for study inclusion

Criteria	Definition
Population^a^	Gay, bisexual or other MSM with a mean age <30 years
Interventions^b^	Online interventions regarding the prevention, care, or treatment of HIV/STBBIs.
Comparisons	No or other HIV/STBBIs prevention approaches.
Outcomes	All outcomes associated with the intervention assessment.
Study Designs	Experimental, quasi‐experimental or pre‐ and post‐ test study design with available follow‐up data.

STBBI, sexually transmitted and blood‐borne infections; MSM, men who have sex with men.

a Studies were included if they had a sample comprising ≥50% gbMSM. Studies that included a mix of gbMSM and other key populations at risk of HIV were only included if they reported one or more primary outcomes separately for gbMSM.

b Interventions included Internet‐enabled apps, webpages and/or social media. This also included interventions that users could use on Internet‐enabled devices such as mobile smartphones, handheld tablet computers (e.g. iPads), laptops and/or desktop computers. We did not include mHealth (i.e. mobile‐based) interventions that did not feature an Internet‐based component for the end‐user (e.g. SMS text messaging interventions).

### 2.3 Data extraction, analysis, and quality assessment

Titles and abstracts of retrieved articles were screened to identify studies that potentially met our inclusion criteria. Full texts of all potentially eligible articles were retrieved by co‐author MK and independently assessed for full inclusion criteria by two review authors (MK and RK). Disagreement or uncertainty between the review authors was resolved through further discussion at weekly team meetings. Each study included was coded by two reviewers for study characteristics (e.g. study date and location), participant characteristics (e.g. target population, age, ethnicity), intervention characteristics (e.g. components, delivery method, duration, setting, theoretical framework), and outcomes (e.g. outcomes measured, main findings). Extracted data were summarized across included studies with respect to: participants and characteristics of studies; interventions and effects; and differential effects in outcomes across participant subgroups.

Risk of bias was assessed using the Cochrane risk of bias instrument for randomized controlled trials (RCTs) 26 and the modified Newcastle Ottawa scale for non‐randomized studies 27. For RCTs, studies were examined for selection bias, performance bias, detection bias, attrition bias, reporting bias, and other potential sources of bias. RCTs were considered at high risk of bias when at least one item was assessed as high risk of bias. For non‐randomized studies, evaluations were made for selection bias, comparability, and outcome assessment.

## 3 Results

**Figure 1:**
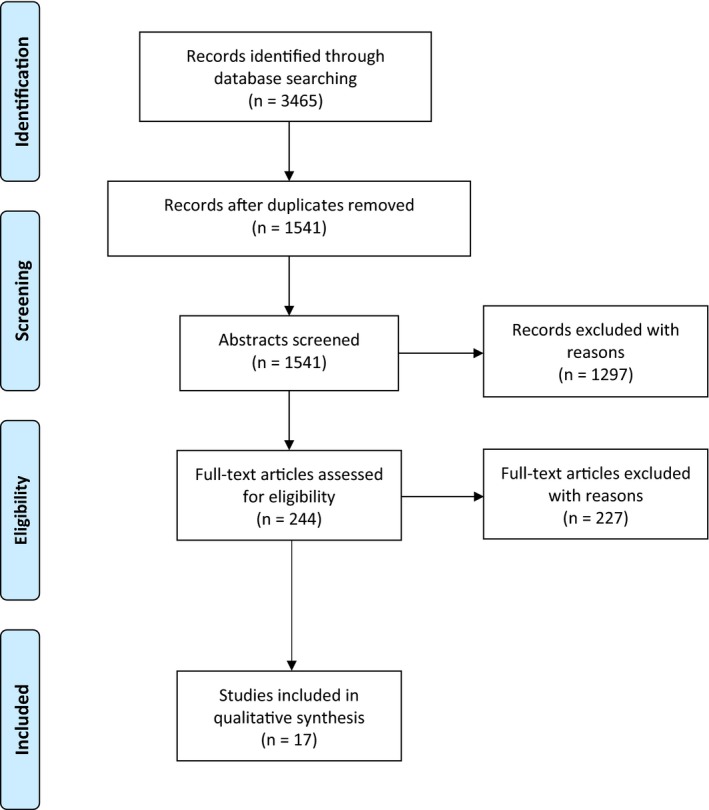
Flow diagram of the selection and review process.

Our search strategy identified a total of 3465 eligible records that were screened for inclusion in the study. Abstract and full‐text screening resulted in a total of 17 included articles 28, 29, 30, 31, 32, 33, 34, 35, 36, 37, 38, 39, 40, 41, 42, 43, 44. A summary of the article collection process is presented in the Preferred Reporting Items for Systematic Reviews and Meta‐Analyses (PRISMA) Flow Diagram in Figure 1. A total of 27 articles were excluded because the mean sample age was ≥30 years 45, 46, 47, 48, 49, 50, 51, 52, 53, 54, 55, 56, 57, 58, 59, 60, 61, 62, 63, 64, 65, 66, 67, 68, 69, 70, 71.

### 3.1 Participants and characteristics of studies

Twelve studies used a randomized control trial design 28, 29, 30, 31, 32, 33, 34, 35, 37, 38, 41, 43, with the remaining five using a pre‐ and ‐poststudy design 36, 39, 40, 42, 44. The 17 studies included 4669 participants at baseline, with sample sizes ranging from 41 to 921 with a median sample size of 130. Twelve studies described theory‐based interventions, including information motivational behavioural theories 29, 32, 35, 39, 40 and integrated behavioural models 33, 38, 44. The remaining theory‐based interventions used other cognition‐based approaches, including the theory of planned behaviour 34, fear‐appeal approach 43, the health‐belief model 31 and social cognitive theory 28, 34. Thirteen studies were conducted in high‐income settings, including 12 from the United States 28, 29, 32, 33, 34, 35, 37, 38, 39, 40, 42, 44 and one from Hong Kong 30. The remaining four were from middle‐income countries, including Thailand 36, Peru 31, 41 and China 43. Full details on the characteristics of study participants are reported in Table 2.

**Table 2:** Characteristics of participants included in a systematic review of online STI/HIV interventions for young gay, bisexual and other MSM

Author (year)	Sample population	Study design/location	Ethnicity	Sexual orientation	HIV status	Inclusion criteria
Bowen et al. (2007)	N = 90 Age: Mean (SD) = 29.02 (9.61)	RCT/USA	18.8% Non‐White; 81.2% White	91.1% Gay; 8.9% Bisexual	100% HIV‐	≥18 years old; Had sex with a man in the last 12 months; Live in a rural area
Bowen et al. (2008)	N = 475 Age: Over 50% <30	RCT/USA	79% Non‐Hispanic White; 9% Hispanic; 12% other	85% Gay; 14% Bisexual; 1% Heterosexual	100% HIV‐	≥18 years old; Had sex with a man in the last 12 months; Live in a rural area
Lau et al. (2008)	N = 280 Age: Over 50% between 20 to 30	RCT/Hong Kong	99.3% Chinese 0.7% Non‐Chinese	NR	NR	≥18 years old; Hong Kong residents; Able to read Chinese; Male persons who had engaged in either oral or anal sex with another man in the past six months; Regular Internet users
Blas et al. (2010)	N = 459 Age: Mean (Range) = 26 (18 to 61)	RCT/Peru	NR	66.4% Homosexual; 33.6% Bisexual	100% HIV‐	≥18 years old; Had sex with men; Resident of Lima, Peru; Not have tested for HIV during the last year; Have an email address; HIV‐
Carpenter et al. (2010)	N = 112 Age: Mean (SD) = 26.3 (5.7)	RCT/USA	15.2% Hispanic/Latino; 6.3% African American; 5.4% Asian American; 0.9% Hawaiian/Pacific Islander; 7.1.% Native American; 80.4% White; 2.7% Other	NR	83.9% HIV−; 16.1% Unknown status	18 to 39 years old; HIV status was negative or unknown; Had engaged in unprotected sex (oral or anal) with a man within the last three months; Had access to a Windows‐based computer with audio capabilities, Internet service, and Internet Explorer; Were willing to provide an active email address for study‐related contact; Could read and understood English; Resided in the US; Had not participated in another psychosocial HIV intervention study in the past year.
Hightow‐Weidman et al. (2012)	N = 50 Age: Mean (Range) = 23.7 (19 to 30)	RCT/USA	100% Black	62% Gay; 30% Bisexual; 8% Other	42% HIV+	18 to 30 years old; Men who had sex with another man in the last 12 months; Self‐identified as Black/African‐American
Christensen et al. (2013)	N = 921 Age: Mean (SD) = 21.3 (1.7)	RCT/USA	74.5% White/Caucasian 14.2 Latino/Hispanic 12.7% Black/African American	76.7% Gay/Homosexual 13.1% Bisexual 10.2% Other	100% HIV−	18 to 24 years old; Engaged in UAI with a non‐primary male partner during the past three‐month; Had a prior HIV‐negative test result; Lived in the United States
Mustanski et al. (2013)	N = 102 Age: Mean (SD) = 21.3 (1.8)	RCT/USA	47% White‐Latino; 26% White‐Non‐Latino; 12% African American; 16% Other	82.3% Gay/Homosexual; 17.7% Bisexual/Other	100% HIV−	18 to 24 years old; Male birth sex and gender identity; HIV‐; Had sex with a male in the prior three months; Had at least one act of unprotected anal sex in the prior three months; Not currently in an exclusive/monogamous relationships lasting longer than 12 months; Able to read at an eighth grade level; Accessed the Internet at least several times in the past month.
Kasatpibal et al. (2014)	N = 162 Age: Mean (SD) = 23.7 (6)	Pre‐ and post‐ test design (Without a comparison group)/Thailand	100% Thai	NR	100% HIV−	Willingness to disclose their sexual orientation to the researchers; Capable of using a computer and the Internet; Having access to a computer and the Internet; Being willing to participate in the research.
Mustanski et al. (2014)	N = 803 Age: Median (IQR): 23 (13)	RCT/USA	77.5% White; 14.8% Hispanic/Latino; 1.1. Black; 6.6% Other	95.1% Homosexual/Gay; 3% Bisexual; 1.9% Other	100% HIV− or Unknown status	≥18 years old; Male sex; Had sex with a man in one's lifetime
Bauermeister et al. (2015)	N = 130 Age: Mean (SD) = 21 (2.23)	RCT/USA	65.6% White, 19.5% Black, 9.4% Latino, 7.8% Middle Eastern, and 6.3% Asian/Pacific Islander	83.8% Gay 14.6% Bisexual 1.6% Heterosexual/Queer	70.8% HIV−	15 to 24 years old; Self‐identify as cis‐male; Reside in the five counties included in the larger Southeast Michigan region; Had sex with a male partner in the prior six months
Lelutiu‐Weinberger et al. (2015)	N = 41 Age: Mean (SD) = 25 (3.22)	Pre‐ and post‐ test design (Without a comparison group)/USA	17.1% Black; 22% Latino; 53.7% White; 7.3% Other	85.4% Gay; 12.2% Bisexual; 2.4% Uncertain	100% HIV− or Unknown status	18 to 29 years old; Born and self‐identified as male; Negative or unknown HIV status; Used drugs – specifically cocaine, methamphetamine, or ecstasy – on at least five of the past 90 days; Had at least one incident of condomless anal sex with an HIV‐positive or status‐unknown main partner, or casual partners of any HIV status in the past 90 days; or, had used the aforementioned drugs with an instance of condomless anal sex meeting the above criteria.
Mustanski et al. (2015)	N = 107 Age: Mean (SD) = 17.9 (1.4)	Pre‐ and post‐ test design (Without a comparison group)/USA	76.6% White; Latino 14.9%; 0.9% Black; 7.4% other	76.6% Gay; 9.3% Bisexual; 6.5% Queers/Unsure	100% HIV− or Unknown status	16 to 20 years old; Identified as LGBT or queer or reported same‐sex attraction or behaviours; Lived in the United States; Engaged in a romantic relationship of any duration with someone of the same biological sex
Young et al. (2015)	N = 556 Age: Mean (SD) = 28.9 (7.9)	RCT/Peru	19.6% White; 2.3% Black; 69.8% Mixed	76.3% Homosexual; 19.1 Bisexual; 4.7% Other	100% HIV− or Unknown status	≥18 years old; Male; Sex with a man in the past 12 months; Living in the Greater Lima Metropolitan area; HIV‐/serostatus unknown; Had a Facebook account or willing to create one
Huang et al. (2016)	N = 122 Age: 65% were 18 to 30	Pre‐ and post‐ test design (Without a comparison group)/USA	14% Black/African American; 86% Hispanic/Latino	NR	100% HIV− or Unknown status	≥18 years old; Self‐identified as Black/African American or Hispanic/Latino MSM.
Lau et al. (2016)	N = 396 Age: 78.8% were 18 to 30	RCT/China	100% Chinese	80.7% Homosexual; 19.2% Bisexual; 4.8% Other	100% HIV− or Unknown status	≥18 years old; Male; Had anal intercourse with at least one man in the last month; Had visited some gay websites at least once per week in the last month; Agreeing not to disseminate the intervention materials to others; Showing ability to go through the online procedures at home; Online HIV prevention naive
Solorio et al. (2016)	N = 50 Age: Mean (SD) = 25 (3)	Pre‐ and post‐ test design (Without a comparison group)/USA	100% Latino	69.4% Homosexual; 20.4% Bisexual/Other; 10.2% Straight	100% HIV− or Unknown status	18 to 30 years old; Self‐report a Latino heritage (e.g. born in a Latin American country); Speak Spanish; Biological male; Report having sex with men in past 12 months; HIV− status

RCT, randomized controlled trials; UAI, unprotected anal sex; NR, not reported.

### 3.2 Inclusion criteria within each study

Eight studies included men 18 and over 28, 29, 30, 31, 37, 41, 42, 43, one ages 15 to 24 38, two ages 18 to 24 34, 35, two ages 18 to 30 33, 44, one ages 16 to 20 40, one ages 18 to 29 39, one ages 18 to 39 32 and one did not report age as being an inclusion criterion 36. Five studies included men who reported having had sex in the past 12 months 28, 29, 33, 41, 44, three in the last six months 30, 32, 34, 35, 38, 39, four in the last three months 32, 34, 35, 39 and one in the last month 43. Two included those who reported ever having had sex with a man 31, 37, one included those who identified as MSM 42, and one included men who had previously been in a “romantic relationship” with someone of the same sex 40. Six studies 32, 34, 35, 39, 41, 44 included only those who were either HIV negative or status “unknown;” the remaining 11 studies 28, 29, 30, 31, 33, 36, 37, 38, 40, 42, 43 did not report serostatus as being an inclusion criterion.

### 3.3 Study quality

Of the non‐randomized studies, three were assessed as high quality 36, 39, 40 and two as low quality 42, 44. Of the RCTs, six were assessed as having a high risk of bias 31, 34, 35, 37, 41, 43 and six as having an uncertain risk 28, 29, 30, 32, 33, 38. Further details on the risk of bias are reported in Tables 3 and 4.

**Table 3:** Quality assessment of non‐randomized studies using the modified Newcastle Ottawa Scale

Author (year)	Selection				Comparability	Outcome			Assessment
	Representativeness	Sample size	Ascertainment of exposure	Non‐respondents	Comparable subjects	Assessment of outcome	Sufficient follow‐up	Total Score	
Kasatpibal et al. (2014)	0	1	1	0	2	0	1	5	High quality
Lelutiu‐Weinberger et al. (2015)	0	0	1	1	2	0	1	5	High quality
Mustanski et al. (2015)	0	1	1	1	2	0	0	5	High quality
Huang et al. (2016)	0	1	1	0	1	0	0	3	Low quality
Solorio et al. (2016)	0	0	1	0	1	0	1	3	Low quality

Studies were considered high quality if they scored above median (i.e., four points).

**Table 4:** Quality assessment of randomized controlled trials using the Cochrane risk of bias tool

Author (Year)	Selection bias		Performance bias	Detection bias	Attrition bias	Reporting bias	Other bias	Assessment
	Random sequence generation	Allocation concealment	Blinding of participants and personnel	Blinding of outcome assessment	Incomplete outcome data	Selective reporting	Other sources of bias	
Bowen et al (2007)	Low	Low	Not applicable	High	Low	Unclear	Low	Unclear Risk of Bias
Bowen et al (2008)	Unclear	Low	Not applicable	High	Low	Low	Low	Unclear Risk of Bias
Lau et al (2008)	Unclear	Low	Not applicable	High	Low	Low	Low	Unclear Risk of Bias
Blas et al (2010)	Low	Low	Not applicable	High	Low	Low	Low	High Risk of Bias
Carpenter et al (2010)	Low	Low	Not applicable	High	Unclear	Low	Low	Unclear Risk of Bias
Hightow‐Weidman et al (2012)	Unclear	Low	Not applicable	High	Low	Low	Low	Unclear Risk of Bias
Christensen et al (2013)	Low	Low	Not applicable	High	High	Low	Low	High Risk of Bias
Mustanski et al (2013)	Low	Low	Not applicable	High	Low	Low	Low	High Risk of Bias
Mustanski et al (2014)	Low	Low	Not applicable	High	High	Low	Low	High Risk of Bias
Bauermeister et al (2015)	Unclear	Low	Not applicable	High	Low	Low	Low	Unclear Risk of Bias
Young et al (2015)	Low	Low	Not applicable	High	Low	Low	Low	High Risk of Bias
Lau et al (2016)	Low	Low	Not applicable	High	Low	Low	Low	High Risk of Bias

‘Low’ in all Domains would place a study at ‘Low Risk of Bias’; ‘High’ in any of the Domains would place a study at ‘High Risk of Bias’; ‘Unclear’ in any of the domains would place the study at ‘Unclear Risk of Bias’

### 3.4 Interventions and effects

All but one study 30 reported a statistically significant effect on one or more outcomes. Of the 17 articles, all focused on behavioural and/or knowledge outcomes at the individual level in order to address HIV/STBBIs in the following intervention categories: 1 reduction of risky sexual behaviours (e.g. condomless sex) via knowledge acquisition and/or attitude change; and 2 testing promotion interventions. One trial 33 assessed an existing “live” intervention in dissemination phase (a website called *healthMpowerment.org*); the remaining 16 were at the “proof‐of‐concept” stage (i.e. at a stage seeking to determine whether an intervention is sufficiently promising to develop and scale). Two reported using tailored interventions (e.g. interventions with the capacity to refine to the level of the individual user) 33, 38, while the remaining used targeted approaches (i.e. focused at the group level, such as at “MSM” or “gay men”). Further details on each intervention, including study limitation and main findings, are reported in Table 5.

**Table 5:** Characteristics of online HIV/STI‐related interventions for young gbMSM*

Author (year)	Intervention description/name	Intervention targets/primary outcomes	Theoretical basis	Main findings	Limitations
Bowen et al. (2007)	Two 20‐minute modules; first module discussing HIV testing, living with HIV, treatment issues and routes of infection; second module focused on safe sex options, condom types and correct condom application. Intervention Name: NR	Reduce HIV‐related risk behaviour; HIV prevention communication; Condom use	SCT	At 1‐week follow‐up (Intervention vs. control): *↑HIV/AIDS knowledge (*p *<* *0.05); *↑Safe sex communication (*p *<* *0.05); *↑Safe sex assertiveness (*p *<* *0.05); *↑Condom use (*p *<* *0.05); *↑Insisting on safe sex (*p *<* *0.05); * Changes were not maintained at 2‐week follow‐up (*p *>* *0.05).	Lack of sufficient time to examine behaviour change; Small sample size; Participants were mostly gay‐identified and White; The two different recruiting methods (face‐to‐face and Internet‐based) may have an effect on outcomes.
Bowen et al. (2008)	The programme included online recruiting, three intervention modules, each with two sessions, online questionnaires. Participants were randomly assigned to one of six module orders and data were collected automatically at pre‐test and after each module. Intervention Name: NR	Reduce HIV‐related risk behaviour; HIV prevention communication; Condom use	IMB	At 2‐week follow‐up (Post‐test vs. pre‐test): * Data supports the feasibility and acceptability of the programme as demonstrated by good retention and rapid programme completion; * Knowledge, self‐efficacy, outcome expectancies and motivation increase in a dose–response fashion (*p *<* *0.001); * Post‐intervention behaviour changes included reduced anal sex and significant increases in condom use (*p *<* *0.01).	Lack of an intervention control group and longer term follow‐up; Limited generalizability due to the nature of the Internet itself; Increased connection speeds and band widths may result in a specific intervention being technologically obsolete in six months.
Lau et al. (2008)	Participants in the intervention group received some visually appealing and professionally designed, educational, email, graphical messages that were related to STD/HIV prevention on a bi‐weekly basis. The control group only received some educational materials. Intervention Name: NR	HIV‐related prevention service utilization; Safe sex practices; Improved sexual behaviours	NR	At 6‐month follow‐up (Intervention vs. control): * No significant improvement in terms of HIV knowledge/perceptions and behaviours (*p *>* *0.1); * Standard, multiple emails with standardized prevention messages may not be effective.	High rates of loss to follow‐up (approximately 40%)
Blas et al. (2010)	5‐minute long videos to incorporate ways to overcome the following different reasons why MSM do not get tested for HIV versus text‐based intervention motivating HIV testing. Intervention Name: NR	Intention to get tested for HIV	HBM	At 4‐month follow‐up (Video‐based intervention vs. text‐based intervention): * Among non‐gay identified MSM participants: Higher likelihood of the reporting of intentions of getting tested for HIV in the next month (RR=2.77; 95% CI: 1.42 to 5.39) and more likely to make an Internet appointment (RR=1.48; 95% CI: 1.13 to 1.95) and to attend the clinic for testing. * Among gay‐identified MSM participants: No significant difference in the reporting of intentions of getting tested for HIV in the next month (RR= 1.54; 95% CI: 0.74 to 3.20) * No significant difference in the reporting of intentions of getting tested for HIV within the next six months among participants from the video‐based intervention and the text‐based intervention in both groups (*p *>* *0.05)	Limited representativeness of the MSM population; Biased sample in terms of educational background and age; Unclear compliance with the interventions
Carpenter et al. (2010)	Participants were randomly assigned to complete either the experimental intervention (1.5 to 2 hours tutorials) or a control intervention (stress reduction training programme) that was not specifically focused on HIV risk. Intervention Name: NR	HIV/STI risk reduction; Increase knowledge of risk factors; Provide skills training for safer behaviour; Increasing motivation for behaviour change	IMB	At 3‐month follow‐up (Intervention vs. control): *↓Numbers of unprotected acts with risky partners for AI, IAI, IOI, and ROI (*p *<* *0.01), but not RAI (*p *=* *0.2); * No significant differences between the groups on ratings of the intervention's ease of use, attractiveness, and enjoyability (*p *>* *0.05).	Low rates of participation by minorities and those of lower socio‐economic status; The brevity and short‐term nature of the intervention; Intervention was delivered remotely (i.e. there is no reliable information about “dosage” and no assurance that the intervention was completed as intended)
Hightow‐Weidman et al. (2012)	Participants were randomly assigned to either the intervention website (Spend at least 30 minutes on the site weekly for four weeks) or a control group. Intervention Name: HealthMpowerment Intervention status: Dissemination phase (at time of study; currently inactive)	Safe sex promotion; Intentions for condom use; Increase HIV/AIDS knowledge	IMB	At 3‐month follow‐up (Intervention vs. control): * ↑Behavioural intentions to use condoms and engage in preparatory condom use behaviours (*p *=* *0.1); *↓Mean scores on the CES‐D scale at the one‐month follow‐up (*p *=* *0.5); * HMP is relevant to the prevention needs of young Black MSM; * Acceptability and feasibility of delivering this prevention programme	Unlike the HMP website, participants in the control condition may have visited different combinations of websites to different extents, and the assumption that they were a uniform group may not be a valid one; Limited generalizability due to the convenience nature of the sample
Christensen et al. (2013)	The intervention used a web‐delivered downloadable simulation game to reduce and assess shame and UAI versus a wait‐listed control group. Intervention Name: SOLVE	Shame reduction; UAI reduction	TPB & SCT	At 3‐month follow‐up (Intervention vs. control): *↑ shame reduction (*p *<* *0.001); *↓ risky sexual behaviour (*p *>* *0.05);	Glitches internal to the game (e.g. Some participants would not download an executable file or others could not play the game given hardware and software configurations; Financial constraints precluded developing characters other than Black, White or Latino, making the game potentially less suitable for other MSM; Low retention rate (69%)
Mustanski et al. (2013)	The Intervention involved seven modules completed across three sessions that were done at least 24 hours apart that in total took two hours to complete. The control condition included HIV information that was available at the time on many existing websites. Intervention Name: Keep It Up!	Safe sex promotion; Increasing HIV knowledge; Improving attitudes towards HIV risk and prevention	IMB	At 12‐week follow‐up (Intervention vs. control): * KIU! intervention can be delivered online safely and with excellent participant engagement; *44% lower rates of unprotected anal sex acts (*p *< 0.05) * No significant difference (*p *> 0.1) in intentions to use condoms, condom errors, condom failures, erection loss, and total sex partners.	Participants completed it under highly controlled conditions, including having study staff provide reminder emails and phone calls and providing participant incentives; All outcomes were measured using self‐report, which are prone to recall and social desirability bias; The self‐efficacy and decisional balance measures had low internal reliability; A relatively small sample
Kasatpibal et al. (2014)	Intervention included logging into the website for four months (one‐group pre‐test and post‐test design). The knowledge test and the HIV‐prevention practices questionnaires were given again. Intervention Name: NR	Increase HIV Knowledge; Decrease HIV‐related risk behaviour	NR	At 4‐month follow‐up (Post‐test vs. pre‐test): *↑Average score of HIV‐prevention knowledge (*p *= 0.000); *↑Average score of practicing HIV prevention (*p *= 0.000); * No significant difference (*p *> 0.05) in frequently changing partners, receiving payment for engaging in receptive anal intercourse without a condom, and having group oral sex without wearing a condom, or without changing the condom when switching partners * Internet‐based instruction was effective in improving HIV‐prevention knowledge and practices among MSM	Relatively small number of MSM in one area of Thailand; Data on sexual behaviours were self‐reported; Participants were volunteers who were willing to identify themselves as MSM, had access to the Internet, and were willing to attend meetings and complete questionnaires.
Mustanski et al. (2014)	Participants were randomly assigned to view informational messages about four prevention options (PrEP, nPEP, rectal microbicides, and condoms). Intervention Name: NR	Condom use promotion	NR	At follow‐up (Comparison between different interventions): * The number of HIV prevention messages did not produce differential attitudes and intentions regarding condoms, nor did it produce changes in attitudes towards unprotected sex (*p *> 0.05); * Receiving multiple messages was associated with greater intentions to use PrEP and nPEP (*p *< 0.01), but not rectal microbicides (*p *> 0.05).	Limited generalizability to individuals who do not use the Internet, social networking sites, or do not respond to advertisements on such sites; African‐Americans are underrepresented in the sample; It is not clear whether receiving these messages alone affected participants’ condom use intentions; Exposure to related information before the intervention were not measured
Bauermeister et al. (2015)	Randomized participants completed a baseline assessment and shown a test‐locator condition (control) or a tailored, personalized site (treatment). Intervention Name: Get Connected!	Promote HIV/STI testing	IBM	At 30‐day follow‐up (Intervention vs. control): * High acceptability among YMSM; *30 participants reported testing, 22 of whom completed the treatment condition (*p *> 0.05). * Of 104 who answered the 30‐day follow‐up, 32 reported making an appointment to get tested for HIV or STIs (*p *> 0.1).	Small sample size; Short follow‐up period; Two competing interventions were evaluated without a no‐treatment control group
Lelutiu‐Weinberger et al. (2015)	Participants completed up to eight‐one‐hour motivational interviewing and cognitive behavioural skills‐based online live chat intervention sessions Intervention Name: MiCHAT	Reduce UAI; Reduce substance use	IMB	At 3‐month follow‐up (Post‐test vs. pre‐test) *↓Days of drug (*p *= 0.08) and alcohol use (*p *= 0.07) in the past month *↓Instances of anal sex without a condom (*p *= 0.04) and number of anal sex without a condom under the influence of drugs (*p *< 0.001) in the past month; *↑Knowledge of HIV‐related risks (*p *= 0.01) and knowledge of the deleterious effects of substance use (*p *= 0.05) * No significant difference in motivation to reduce condomless anal sex acts and drug use or behavioural efficacy skills as well as no reduction in depressive symptoms (*p *= 0.19) and gay‐related concealment stigma (*p *= 0.18)	Small sample size and low power; Lacking a control group; Small‐to‐moderate effect sizes; Short follow‐up period
Mustanski et al. (2015)	The QSE intervention consisted of an introduction and five intervention modules that followed a common sequencing. Each module ended with a quiz about the presented materials, which was used as a didactic method to reinforce learning. Intervention Name: Queer Sex Ed	Sexual orientation identity and self‐acceptance; Sexual health knowledge increase * Safe sex communication * Safe sex promotion	IMB	At 2‐week follow‐up (Post‐test vs. pre‐test): *15 of the 17 outcomes were found to be significantly improved (*p *< 0.05); * Effect sizes ranged from small for sexual orientation (e.g. internalized homophobia) and relationship variables (e.g. communication skills) to moderate for safer sex outcomes (e.g. contraceptive knowledge); * Feasibility, acceptability, and initial efficacy of QSE	Short follow‐up; Self‐reported outcomes; Relatively low participation of African‐American youth
Young et al. (2015)	Participants were randomly assigned to join private intervention (be HIV prevention mentors to participants via Facebook groups) or control groups (received an enhanced standard of care) on Facebook for 12 weeks. Intervention Name: HOPE	* HIV testing promotion	NR	At 12‐week follow‐up (Intervention vs. control): *↑Request for HIV testing (AOR: 2.79, 95% CI: 1.42 to 5.72) *↑HIV testing (AOR: 2.61, 95% CI: 1.55 to 4.38) * No adverse events were reported and retention was high; *↓Self‐reported engagement in receptive anal sex (*p *> 0.05) * Across conditions, 7 (87.5%) of the eight participants who tested positive were linked to care at a local clinic	Limited findings based on study location and population. Self‐reported outcomes
Huang et al. (2016)	Grindr users who clicked on an advertisement (Posted for six weeks) were directed to our study website, where they were asked to choose out of one of three methods for self‐test delivery: (1) U.S. Postal Service, (2) a Walgreens voucher, or (3) a vending machine. Intervention Name: HIV Self‐Test Programme	* HIV testing promotion	NR	At 2‐week follow‐up (Post‐test vs. pre‐test): * Among 57 survey respondents, 55 (97%) reported that using the self‐test was easy; * Two persons reported testing HIV positive and both sought medical care; * Social networking application self‐ testing promotion resulted in a large number of self‐test requests and has high potential to reach untested high‐risk populations who will link to care if they test positive.	Self‐reported survey responses; No fail‐proof method to verify that survey responses were unique; Limited availability of the vending machine delivery method; Relatively small sample size
Lau et al. (2016)	Participants were approached through three channels: (i) the Internet (ii) gay‐venues (gay bars and gay saunas), and (iii) snowball referrals to watch short videos (5 to 10 minutes) about STD prevention. Control group participants received factual HIV‐related text information. Intervention Name: NR	* Reduce UAI * Safe sex promotion	Fear appeal approach	At 3‐month follow‐up (Intervention vs. control) * Higher score in the STD‐related cognitive plus fear appeal imagery approach assessing fear after the watching the intervention materials (*p *< 0.001); * No statistically significant differences were found across the three groups regarding UAI (*p *> 0.05)	Self‐reported and social desirability biases; Potential Hawthorne effect; Intervention limited to MSM who had access to the Internet; Induced fear might have faded away over time. The follow‐up period of three months was relatively short
Solorio et al. (2016)	The 16‐week campaign included Spanish‐language radio public service announcements (PSAs), a Web site, social media outreach, a reminder system using mobile technology, print materials and a toll‐free hotline. Intervention Name: Tu Amigo Pepe	* HIV testing promotion	IBM	At 2‐month follow‐up (Post‐test vs. pre‐test): *↑ attitudes (*p *= 0.03), beliefs (*p *= 0.01) towards condom use; *↑ attitudes (*p *< 0.001), intentions (*p *= 0.01), norms and self‐efficacy (*p *= 0.004) towards HIV testing with HIV testing rates increasing over time	NR

CES‐D, Center for Epidemiological Studies Depression; URAI, Unprotected Receptive Anal Intercourse; UIAI, Unprotected Insertive Anal Intercourse; UROI, Unprotected Receptive Oral Intercourse; UIOI, Unprotected Insertive Oral Intercourse; UAI, Unprotected Anal Sex; SCT, Social Cognitive Theory; IMB, Information‐Motivation‐Behavioural Skills Model; HBM, Health‐Belief Model; TPB, Theory of Planned Behaviour; IBM, Integrated Behavioural Model; PrEP, Pre‐exposure Antiretroviral Prophylaxis; nPEP, non‐occupational Post‐Exposure Prophylaxis; QSE, Queer Sex Ed; HMP, HealthMpowerment; YMSM, Young Men Who Have Sex with Men; RR, Relative Risk; AOR, Adjusted Odds Ratio; CI, Confidence Interval; NR, Not Reported; gbMSM gay, bisexual and other men who have sex with men.

#### 3.4.1 Reduction of risky sexual behaviour via knowledge acquisition and/or attitude change

Three non‐randomized studies sought to assess risky sexual behaviour change following interventions to improve knowledge and/or attitude change. Kasatpibal *et al*. 2014 36 provided participants with a log‐in code to an Internet site that offered HIV prevention information in the form of texts, pictures, animated cartoons, videos, message boards and exercises. The study reported an increase in HIV knowledge and a decrease in HIV risk practices. Lelutiu‐Weinberger *et al*. 2015 39 provided participants with online counselling sessions via Facebook chat, and the findings indicated a preliminary efficacy for reducing condomless sex, substance use, and their co‐occurrence. Mustanski *et al*. 40 provided participants with five intervention modules that ended with a quiz about the presented materials. The study reported an increase in 15 of 17 attitudinal and behavioural outcomes.

Seven RCTs aimed to change knowledge, attitudes, or behaviours, and included an assessment of behaviour change as a primary or secondary outcomes. Bowen *et al*. 2008 29 conducted an RCT in which they offered six modules of various scenario content (including on topics of HIV prevention, “contexts” of risk and experiences with new and casual partners). They reported a statistically significant change in knowledge, self‐efficacy, and motivation to engage in risk‐reduction practices, in addition to reduced anal sex and significant increases in condom use. Lau *et al*. 2008 30 provided an experimental group with bi‐weekly “visually appealing and professionally designed, educational, email graphical messages” on the topics of HIV/STD prevention, including HIV transmission, correct condom use, HIV testing, relationship and love, and the relationship between drugs and sex. The authors did not find a significant change in risk behaviour and perceptions following intervention and warned that the effectiveness of online interventions should not be taken for granted. Carpenter *et al*. 32 provided an experimental group with a 90‐minute motivational, informational and skills training modules, including interactive materials, multimedia presentation and didactic text, followed by an opportunity to test their knowledge about HIV risk and learn up‐to‐date information. Findings indicated reductions among the experimental group with risky sexual practices with those with the “riskiest” sexual partners (those who reported having partners who were either seropositive or of an “unknown” status), not including condomless receptive anal intercourse. Christenson *et al*. 34 provided a simulation using an avatar that is designed to reduce shame associated with sexual stigma among MSM by allowing participants to view their sexual desires as being “normal.” The study reported reductions in self‐reported feelings of shame among the experimental group, though there was not a direct effect to reductions in risky sexual behaviour at follow‐up. Mustanski *et al*. 35 offered a total of seven online learning modules to participants on a variety of topics that were designed for young MSM upon receiving an HIV‐negative test. Compared to the control group, participants in the experimental arm had a lower rate of unprotected anal sex acts at follow‐up. Lau *et al*. 43 assessed two fear appeal approaches to video‐based interventions (five to ten minutes) – one related to enhancing fear of health implications of contracting an STI and the other related to enhancing fear of the social losses associated with contracting an STI. The study did not find a statistically significant difference in unprotected anal intercourse among the intervention and that of a “factual” text‐based website control. Hightow‐Weidman *et al*. 33 conducted an RCT that provided the experimental group with access to an existing online informational website called *Mpowerment*; key features of the site included tailored live chats with an HIV expert, interactive quizzes, “hook‐up/sex” journals, and HIV/STBBI risk assessment tools. The study reported changes in intention to use condoms and engage in preparatory condom use behaviours, though this was not a condition of the intervention (i.e. both control and experimental groups experienced this); no change in risk behaviour was reported.

The remaining two RCTs aimed to change knowledge and/or attitudes but did not include an assessment of behaviour change following the intervention. Bowen *et al*. 28 conducted an RCT that reported a statistically significant improvement in HIV/AIDS‐related knowledge and safer sex attitudes after delivering two online scenario‐based modules. The intervention consisted of approximately 20‐minute module regarding various scenarios about risk behaviour and an “inexperienced” man's experiences with a risky sexual encounter and the potential of having become infected. Mustanski *et al*. 37 conducted an RCT to measure the effects of HIV prevention messaging videos about multiple biomedical and behavioural HIV prevention methods (including nPEP, PrEP, rectal microbicides, and condoms) and MSM's intentions to use these strategies. The study found that the number of prevention messages did not produce differential attitudes and intentions regarding condoms; however, receiving multiple messages at once was associated with greater intentions to use PrEP and nPEP, but not rectal microbicides.

#### 3.4.2 Testing promotion interventions

Three RCTs assessed testing behaviour change following an online intervention. Blas *et al*. 31 randomized MSM to receive either a traditional public health text‐based intervention (control group) or a five‐minute video‐based HIV testing promotion video (experimental group) targeted to either (i) gay, or (ii) non‐gay MSM. They reported a statistically significant increase in intention to get tested among non‐gay identified MSM following intervention, as well as following through to do so. Young *et al*. 41 conducted an RCT with peer‐leaders creating a Facebook group and inviting participants to join and encouraging them to test throughout the duration of the 12‐week study. Those in the intervention were more likely to test than those in the control. Bauermeister *et al*. 38 tailored the content of an online intervention based on the experimental group's socio‐demographic data, including age, race/ethnicity, sexual identity, relationship status, testing history, sexual behaviour and structural barriers (e.g. Black MSM saw pictures of Black men). While testing practices were higher among the intervention group, this was not statistically significant; however, the difference was clinically meaningful with Cohen's *d *= 0.34, leading the authors to suggest preliminary efficacy.

Two non‐randomized studies assessed testing behaviour following the intervention. Huang *et al*. 42 recruited participants from *Grindr* to receive a self‐test kit (either via a pharmacy rebate code, via the mail or through a vending machine at a local LGBTQ centre). The study found that social network advertising that links users to a self‐test was successful. Solorio *et al*. 44 conducted a multi‐media campaign that included social media outreach and web‐based informational pages to encourage testing among Latino MSM. The study found a significant impact on testing behaviour.

### 3.5 Intervention acceptability

Thirteen studies reported that the interventions were considered acceptable to participants 28, 29, 31, 32, 33, 34, 35, 38, 39, 40, 41, 42, 44, but five of these 32, 33, 41, 42, 44 did not provide details on how these data were collected. The fear appeal intervention 43 was assessed as not being acceptable. Three studies did not report on the acceptability of the intervention. Further details on intervention acceptability are reported in Table 6.

**Table 6:** Acceptability of online HIV/STI‐related interventions for young MSM

Author (date)	Acceptable (yes or no)	Acceptability measurement	Acceptability reasons
Bowen et al. (2007)	Yes	Six‐point Likert‐type scales using five questions	* Interesting intervention * Exciting graphics * Proper length of intervention
Bowen et al. (2008)	Yes	High retention and completion rates	* Multi‐session with a range of foci
Lau et al. (2008)	Not Effective Intervention‐ Not Reported	NA	NA
Blas et al. (2010)	Yes	Five‐point Likert‐type scales using one question	Video content
Carpenter et al. (2010)	Yes	Pilot testing with 21 samples using a range of questions	NR
Hightow‐Weidman et al. (2012)	Yes	Five‐point Likert‐type scales using twenty questions	NR
Christensen et al. (2013)	Yes	NR	* Web‐based simulation game
Mustanski et al. (2013)	Yes	Five‐point Likert‐type scales using eight questions	* Interactivity of the modules * Variety of media used (e.g. video, game, graphics) * Colloquial language * Relevance of scenarios incorporated to the video
Kasatpibal et al. (2014)	Effective Intervention‐ Acceptability Data Not Reported	NA	NA
Mustanski et al. (2014)	Effective Intervention‐ Acceptability Data Not Reported	NA	NA
Bauermeister et al. (2015)	Yes	Seven‐point Likert‐type scales using six questions	* Providing accurate information * Easy to use
Lelutiu‐Weinberger et al. (2015)	Yes	One‐hour phone interview at the end of the survey	* Appropriate duration of sessions and intervention * Relevant content * Non‐judgemental and professional approach/tone
Mustanski et al. (2015)	Yes	Qualitative interviews	* Including information about relationship skills and sexual functioning rather than just providing information about STIs * It was not just scare tactics and what is taught in school‐based sex education * It was fun and they did not feel “talked down to” * It helped make them feel empowered in their sexual health
Young et al. (2015)	Yes	Based on the high retention rate (90%)	NR
Huang et al. (2016)	Yes	Five‐point Likert‐type scales using two questions	NR
Lau et al. (2016)	Not very acceptable	NR	Not very acceptable
Solorio et al. (2016)	Yes	NR	NR

### 3.6 Differential intervention effects

#### 3.6.1 Sexual identity

Measures of sample composition regarding sexual identity were included in thirteen of the studies, with each of these reporting on two or more sexual identities (including gay, bisexual, queer, straight and “other”) 28, 29, 31, 33, 34, 35, 37, 38, 39, 40, 41, 43, 44. The remaining four did not measure and/or report sexual identity 30, 32, 36, 42.

The majority of studies did not report on or examine differential effects by sexual identity. However, two studies comprised a research design that was specifically designed to assess intervention effect by sexual identity. An RCT based in Peru by Blas *et al*. 31 split their study sample between MSM that were “gay‐identified” (including those who identify as “gay” or “*caleta*” – i.e. those who identified as “closeted” or “semi‐closeted”) and “non‐gay‐identified” (including those who identify as heterosexual, bisexual or “*flete*” – i.e. young male prostitutes). Each cluster was split and therefore received both the control and experimental conditions; the intervention – a five‐minute HIV testing health promotion video – was targeted towards either gay‐ or non‐gay‐identified MSM. The authors found a significant difference only among non‐gay identified MSM, thereby theorising that the non‐gay‐identified men were more receptive to interventions that promote HIV testing.

A non‐randomized study by Solorio *et al*. 44 hypothesized that non‐gay‐identified Latino men in Seattle, Washington, would be less likely to be responsive to campaigns that were not targeted specifically towards the Spanish‐speaking Latino community. As such, a multi‐media campaign was targeted to Spanish‐speaking MSM who do not identify as gay and also for those who identify as gay. The study reported that MSM who did not identify as gay were just as likely to seek HIV testing following the intervention as those who identified as gay. The authors considered these results successful as non‐gay‐identified Latino MSM represented a “difficult‐to‐reach” population.

#### 3.6.2 Gender identity

Blas *et al*. 31 reported including both transgender‐ and cisgender‐identified MSM. This study included an intervention arm that was specifically designed for transgender MSM; however, they lacked sufficient power to evaluate the transgender arm (n = 21) and the transgender group was therefore excluded from the analysis. Mustanski *et al*. 40 reported that they included four transgender men; no sub‐analysis was conducted by gender identity. Bauermeister *et al*. 38 reported that transgender men were excluded from their study. The remaining 14 studies did not report the gender identities of participants.

#### 3.6.3 HIV status

Six studies reported samples comprised of only HIV‐negative participants 28, 29, 31, 34, 35, 36, while eight studies reported samples with a composition of HIV‐negative and “unknown” serostatus 32, 37, 39, 40, 41, 42, 43, 44. One study did not report on serostatus 30. “Unknown” serostatus was generally used to refer to participants who had not tested, either previously or recently.

Two studies reported including HIV‐positive participants. An RCT by Hightow‐Weidman *et al*. 33 reported they randomized more HIV‐positive participants into their control group. Unfortunately, due to a low sample size, they were unable to account for serostatus in their analysis. As such, the authors suggest that they may have been unable to detect behaviour change resulting from the intervention because the HIV‐positive participants were likely engaging in condomless sex with sero‐concordant partners. These authors suggest future studies should account for differential effects of HIV status both through statistical controls and a stratified randomized design in order to ensure serostatus differences are sufficiently powered to assess both study conditions (i.e. intervention and control). Bauermeister *et al*. 38 also assessed and reported on HIV serostatus; their study sample included four (3.0% of the sample) who were HIV positive in their HIV/STBBI testing promotion intervention. While they did not assess the differential effects of the intervention by serostatus, they did indicate that all four HIV‐positive participants reported seeing a HIV/STI provider in the past 30 days.

#### 3.6.4 Socio‐economic status

All but one study 43 included one or more measures of socio‐economic status (SES): five measured only educational attainment 29, 31, 34, 37, 40, three measured educational attainment and employment status 35, 38, 42, four measured educational attainment and income 32, 33, 39, 41, one measured employment and income 28, and three measured employment status, income and educational attainment 29, 36, 44. None of the studies reported on differential effects of interventions by SES.

#### 3.6.5 Ethno‐racial characteristics

Among the studies from Asia, one from China 43 and one from Hong Kong reported sample 30 compositions that were entirely Chinese and one study from Thailand reported an entirely Thai sample 36. One study from Peru did not report ethno‐racial identity 31 while another reported a mixed sample composition (mixed, White and Black) 41. One study from the US specifically focused on recruiting Black MSM 33, another Latino MSM 44 and a third both Black and Latino MSM 42, as these populations were specifically identified as being at an elevated risk for HIV/STBBIs. One study 28 from the US reported on ethno‐racial identity as being either “White” or “non‐White.” The remaining eight studies 29, 32, 34, 35, 37, 38, 39, 40 from the US included at least four or more measures for ethno‐racial identities, including (in order of most frequently used to least) White, Latino or Hispanic, Black or African American, “other,” Middle Eastern, Native American, Asian Pacific and Hawaiian Pacific Islander. None of the studies reported on differential effects of interventions by ethno‐racial identity.

## 4 Discussion

Our systematic review of online approaches to address the prevention, care, and/or treatment of HIV/STBBIs among young gbMSM included 12 RCTs and five non‐randomized studies. Sixteen of the studies in our review were “proof‐of‐concept” efficacy trials of interventions not specifically designed for further dissemination; accordingly, the sample sizes were generally small (median: 130 participants). One study assessed a “live,” “real‐world” intervention. All of the studies focused on behavioural or knowledge outcomes at the individual level (e.g. condom use, testing behaviour, and knowledge and/or attitudes about HIV/STBBI risk), and all but one reported a statistically significant effect on one or more primary outcomes. Twelve studies described theory‐based interventions. Twelve were conducted in the United States, with study samples focusing mainly on White, African‐American and/or Latino populations; the remaining were conducted in Hong Kong, Peru, China, and Thailand. Thirteen studies included gay and bisexual men; four studies did not assess sexual identity. Two studies reported including both HIV‐positive and HIV‐negative participants, and all but one study included one or more measure of SES (e.g. income, educational attainment). While most (n = 13) of the interventions included and reported upon measures of intervention acceptability, five of these did not provide details on how this was assessed; the remaining four did not report on intervention acceptability.

The statistically significant changes in one or more primary outcome in all but one of our included studies underscores the promise that online approaches have for addressing HIV/STBBIs among young gbMSM. As such, our review supports previous research 9 suggesting that efforts to change behaviour at the population level may benefit from evidence‐informed online approaches. Nevertheless, as no trial had a low risk of bias for all quality criteria, the promising results need to be interpreted with caution and confirmed in further high‐quality trials. Moreover, there were several limitations associated with the measurements used across the studies included in our review. First, all of the studies focused on behavioural interventions, with no studies assessing the efficacy or effectiveness of other kinds intervention types (e.g. biomedical, structural). Second, although behavioural outcomes (e.g. condom use, HIV testing) resonate with the National HIV/AIDS Strategy for the United States (NHAS) 2015 indicators for young MSM 72, study outcomes were measured and reported in highly variable and inconsistent ways across studies, making it difficult to compare findings across studies and precluding our ability to pool the results. Future studies should seek to use standardized measures whenever possible to assess the effects of online interventions on different outcomes.

The majority of research to date in this area is largely focused on “proof‐of‐concept” and/or “one‐off” interventions that are not sustained beyond the completion of the study. Out of the 17 studies included in our review, only one 33 sought to assess an intervention that was within the dissemination phase (i.e. it was “live” and available to the public while the study was taking place). Moving beyond the “proof‐of‐concept” research phase into the dissemination of interventions in “real‐world” conditions will benefit from including additional implementation measures during all phases of intervention research 73. For example, online HIV/STBBI intervention research should be designed with a variety of implementation‐oriented considerations in mind to systematically identify the factors that will influence intervention scalability (e.g. equitable reach; rate of uptake) among key groups of young gbMSM within and across a variety of settings 16.

We also suggest that future intervention research in this area will benefit from enhanced efforts to assess the effects of various “real‐world” and “live” interventions, (e.g. rather than focusing on “proof‐of‐concept” trials), including risk‐reduction interventions that have been developed and implemented from outside of the health or community‐based sectors. For example, within the private technology sector, *Grindr* recently provided users with the option to disclose their serostatus, viral load (e.g. “undetectable”) and/or use of PrEP on users’ profile pages. Identifying the effects that these kinds of “real‐world” interventions may have on the social and sexual health outcomes of young gbMSM is a critical “next‐step” for intervention research in this area.

Most of the studies included one or more socio‐demographic measures to describe the sample composition (e.g. by SES, sexual identity, serostatus). However, with a few notable exceptions, few reported on the differential intervention effects by SES, sexual or gender identity, ethno‐racial characteristics or HIV serostatus, often due to small sample sizes and sample compositions that were too homogenous. We suggest that future research regarding online sexual health interventions undertake differential analyses, particularly in light of growing evidence that suggests individually‐oriented interventions tend to (re)produce inequalities in health 74, 75. We agree with Hightow‐Leidman *et al*. 33 suggestion that future research designs should – whenever possible – seek to account for differential effects through the use of statistical controls and/or stratified randomized designs, or stepped‐wedge designs for those interventions in the dissemination phase. This may also require additional measures to effectively ensure key differences (e.g. by serostatus, sexual identity, SES) are sufficiently powered to assess differential effects (e.g. within and across study conditions).

We were surprised that few studies focused on evaluating interventions among several “key” populations of young gbMSM, including those living with HIV, transgender gbMSM and those living in low‐income settings. For example, given the ongoing shifts in the field of HIV emphasizing the treatment and prevention needs for those living with HIV, research assessing online HIV/STBBI interventions for HIV‐positive populations seems notably absent 16. Furthermore, out of the 4669 participants included in our review, only 25 participants were reported as identifying as transgender. Finally, to date, no studies assessed online approaches to addressing HIV/STBBI among young gbMSM in low‐income settings. Future research in low‐ and middle‐income settings is needed. Critically, it is important to emphasize that if key groups of MSM are not included in online intervention research, these groups are likely to be excluded from population‐specific interventions to reduce HIV/STBBI risk as they are scaled up to the population level 76.

There was also a tendency for the interventions in our review to be based on the premise that a “one‐size‐fits‐all” approach to intervention delivery can work for all populations of gbMSM, regardless of serostatus, SES, and other circumstances. For example, we were surprised that most interventions employed “targeted” approaches (i.e. at a population's group characteristics), particularly given the public health science in this area that indicates that tailored (i.e. at an individual's characteristics) web‐delivered behaviour change intervention are significantly more effective than non‐tailored websites in achieving behavioural outcomes 77. Indeed, only Hightow‐Leidman *et al*. 33 and Bauermeister *et al*. 38 reported tailoring content and user experience based on user‐specific data profiles (e.g. based on age, ethnicity, sexual identity). Given that interventions that tailor approaches to an individual's specific “profile” (e.g. based on various features of their social positioning) tend to better capture a user's attention, contain less redundant information and overall be more acceptable among users 77, future online intervention development in this area may benefit from developing sophisticated approaches to tailored web‐based service delivery systems.

### 4.1 Limitations

Our review – the first review focusing on online interventions regarding the prevention, care, and/or treatment of HIV/STBBIs among young gbMSM – has several strengths and limitations. First, a limitation of our review is that because of the high‐level of heterogeneity between the different risk‐reduction and testing promotion interventions and measured outcomes, it is not feasible to calculate the pooled effects of the interventions included in our review. Second, while our approach to searching the literature was comprehensive and employed a robust set of search strategies, including the use of multiple databases, potentially relevant studies that are reported in other domains (e.g. technical reports in the grey literature; non‐English peer‐reviewed publications) are not accounted for in our review. Third, because our inclusion criteria required a sample of at least 50% gbMSM under 30 years of age, studies that may have reported relevant findings about online intervention with gbMSM are excluded 45, 46, 47, 48, 49, 50, 51, 52, 53, 54, 55, 56, 57, 58, 59, 60, 61, 62, 63, 64, 65, 66, 67, 68, 69, 70, 71; future analyses of these studies will provide important details about the experiences of older generations of gbMSM with online interventions. Nevertheless, these findings provide a key “first step” in informing both potentially effective strategies and a renewed research agenda regarding the development of evidence‐based online interventions to address HIV/STBBIs among young gbMSM.

## 5 Conclusions

On the basis of our findings, we support a call for more rigor and attention within the creation of study designs that have the capacity to report differential effects within and across population sub‐groups in intervention research 74 in order to unpack the potentially distinctive experiences of particular subgroups of young gbMSM (e.g. lower vs. higher income gbMSM). We also urge researchers in this area to identify the effects of “real‐world,” “live” interventions, including online sexual health programmes that provide a service to the public (e.g. online testing platforms) and/or interventions that are put forth from the private technology sector (e.g. risk‐reduction strategies that are programmed within social and sexual networking apps). Finally, future research must also assess intervention effects among young gbMSM who are transgender, living in low‐income settings and/or who are living with HIV.

## Competing interests

None to declare.

## Authors’ contributions

All co‐authors contributed to the research questions. MK offered technical expertise in developing our search strategy. RK and MK extracted data and conducted the data analysis. RK drafted the first version of the manuscript and received extensive feedback from all co‐authors.

## Funding

This study was funded by the Canadian Institutes of Health Research (145,373). Knight is supported by a Post‐Doctoral Fellowship from the Canadian Institutes of Health Research and the Michael Smith Foundation for Health Research.

## Supporting information


**Table S1.** Quality Assessment of non‐randomized studies using the modified Newcastle Ottawa Scale
**Table S2.** Quality Assessment of randomized controlled trials using the Cochrane risk of bias tool

**Appendix S1.** PRISMA 2009 checklist.

**Appendix S2.** Sample search strategy to search the Medline database via the OVID platform; 15 November 2016.

## Note

1 We define youth as being <30 years, in accordance with emerging trends in the operationalization of young adult age groups (e.g. European Union; Millennial Generation), accounting for a set of secular trends, including delayed transitions into “adulthood” (e.g. leaving parental home; achieving financial independence).
