# Supplementary material for: Online interventions to address HIV and other sexually transmitted and blood‐borne infections among young gay, bisexual and other men who have sex with men: a systematic review
Source: J Int AIDS Soc. 2017 Nov 1;20(3):e25017. doi: 10.1002/jia2.25017 (PMC5810340; doi:10.1002/jia2.25017)
Supplement: Supplementary file 1 — Table S1. Quality Assessment of non‐randomized studies using the modified Newcastle Ottawa Scale Table S2. Quality Assessment of randomized controlled trials using the Cochrane risk of bias tool [file JIA2-20-e25017-s001.docx]

**Supplemental Table 1. Quality Assessment of Non-randomized Studies Using the Modified Newcastle Ottawa Scale**

|  | **Selection** | | | | **Comparability** | **Outcome** | | **Total Score** | **Assessment** |
| --- | --- | --- | --- | --- | --- | --- | --- | --- | --- |
| **Author (year)** | **Representativeness** | **Sample size** | **Ascertainment of exposure** | **Non-respondents** | **Comparable subjects** | **Assessment of outcome** | **Sufficient follow-up** |  |  |
| Kasatpibal et al. (2014) | 0 | 1 | 1 | 0 | 2 | 0 | 1 | 5 | High Quality |
| Lelutiu-Weinberger et al. (2015) | 0 | 0 | 1 | 1 | 2 | 0 | 1 | 5 | High Quality |
| Mustanski et al. (2015) | 0 | 1 | 1 | 1 | 2 | 0 | 0 | 5 | High Quality |
| Huang et al. (2016) | 0 | 1 | 1 | 0 | 1 | 0 | 0 | 3 | Low Quality |
| Solorio et al. (2016) | 0 | 0 | 1 | 0 | 1 | 0 | 1 | 3 | Low Quality |

Note: Studies were considered high quality if they scored above median (i.e., four points)

**Supplemental Table 2. Quality Assessment of Randomized Controlled Trials Using the Cochrane Risk of Bias Tool**

| **Author (Year)** | **Selection bias** | | **Performance bias** | **Detection bias** | **Attrition bias** | **Reporting bias** | **Other bias** | **Assessment** |
| --- | --- | --- | --- | --- | --- | --- | --- | --- |
|  | **Random sequence generation** | **Allocation concealment** | **Blinding of participants and personnel** | **Blinding of outcome assessment** | **Incomplete outcome data** | **Selective reporting** | **Other sources of bias** |  |
| Bowen AM et al (2007) | Low | Low | Not Applicable | High | Low | Unclear | Low | Unclear Risk of Bias |
| Bowen AM et al (2008) | Unclear | Low | Not Applicable | High | Low | Low | Low | Unclear Risk of Bias |
| Lau JTF et al (2008) | Unclear | Low | Not Applicable | High | Low | Low | Low | Unclear Risk of Bias |
| Blas MM et al (2010) | Low | Low | Not Applicable | High | Low | Low | Low | High Risk of Bias |
| Carpenter KM et al (2010) | Low | Low | Not Applicable | High | Unclear | Low | Low | Unclear Risk of Bias |
| Hightow-Weidman LB et al (2012) | Unclear | Low | Not Applicable | High | Low | Low | Low | Unclear Risk of Bias |
| Christensen JL et al (2013) | Low | Low | Not Applicable | High | High | Low | Low | High Risk of Bias |
| Mustanski B et al (2013) | Low | Low | Not Applicable | High | Low | Low | Low | High Risk of Bias |
| Mustanski B et al (2014) | Low | Low | Not Applicable | High | High | Low | Low | High Risk of Bias |
| Bauermeister J et al (2015) | Unclear | Low | Not Applicable | High | Low | Low | Low | Unclear Risk of Bias |
| Young SD et al (2015) | Low | Low | Not Applicable | High | Low | Low | Low | High Risk of Bias |
| Lau J et al (2016) | Low | Low | Not Applicable | High | Low | Low | Low | High Risk of Bias |

Note: ‘Low’ in all Domains would place a study at ‘Low Risk of Bias’; ‘High’ in any of the Domains would place a study at ‘High Risk of Bias’; ‘Unclear’ in any of the domains would place the study at ‘Unclear Risk of Bias’
