# Supplementary material for: Online interventions to address HIV and other sexually transmitted and blood‐borne infections among young gay, bisexual and other men who have sex with men: a systematic review
Source: J Int AIDS Soc. 2017 Nov 1;20(3):e25017. doi: 10.1002/jia2.25017 (PMC5810340; doi:10.1002/jia2.25017)
Supplement: Supplementary file 3 — Appendix S2. Sample search strategy to search the Medline database via the OVID platform; 15 November 2016. [file JIA2-20-e25017-s003.docx]

**S1. Sample Search Strategy to Search the Medline Database via the OVID Platform; Nov. 15, 2016**

| **No.** | **Search Terms** |
| --- | --- |
| 1 | exp homosexuality/ OR homosexuality male/ OR exp bisexuality/ OR (Homosexual* OR bisexual* OR (men who have sex with men OR gay OR Men Having Sex with Men OR (Male adj (prostitut$ OR 'sex worker')) OR ((homosexual$ OR gay) adj2 (men OR man OR male OR males)) OR MSM* OR MSMW)).ti,ab. |
| 2 | exp HIV/ OR exp HIV Infection/ OR exp Acquired Immune Deficiency Syndrome OR exp Human Immunodeficiency Virus/ OR exp Sexually Transmitted Diseases OR (HIV OR HIV infect* OR human immune?deficiency virus OR human immuno?deficiency virus OR human immunedeficiency virus OR human immun* deficiency virus OR acquired immune?deficiency syndrome OR acquired immuno?deficiency syndrome OR acquired immunedeficiency syndrome OR acquired immun* deficiency syndrome).ti,ab. |
| 3 | (gonorrhea* OR gonnococcal infection* OR gonorrhoea OR Neisseria gonorrhoeae OR (chlamydia OR chlamydia infection* OR Chlamydia trachomatis) OR (syphilis OR Treponema pallidum) OR (papillomavirus OR human papillomavirus OR HPV) OR (herpes genitalis OR genital herpes OR Herpes Simplex Virus Genital Infection OR Genital Herpes Simplex OR Herpes Simplex Virus OR HSV) OR (STI* OR STD* OR sexually transmitted infection* OR sexually transmitted disease* OR STBBI* OR "sexually transmitted and blood-borne infection*") OR (trichomoniasis OR trichomonas infection* OR Trichomonas vaginalis) OR (sexual health OR sex* behavior OR oral sex OR anal sex OR anal intercourse OR safe sex OR protected sex OR responsible sex OR unsafe sex OR unprotected sex OR high?risk sex OR risky sex)).ti,ab. |
| 4 | (exp hepatitis B/ OR exp Hepatitis B virus/) OR (hepatitis B OR HBV OR hepabivirus OR Chronic hepatitis B). ti,ab. |
| 5 | (exp hepatitis C/ OR exp Hepatitis C virus/) OR (hepatitis C OR HCV OR hepacivirus OR Hepatite OR VHC). ti,ab. |
| 6 | 2 OR 3 OR 4 OR 5 |
| 7 | (promot* OR prevent* OR interven* OR program* OR implement* OR evaluat* OR scal* up OR effectiveness).ti,ab. |
| 8 | Exp online systems OR exp internet OR exp Computer Systems OR exp Medical Informatics OR exp Telemedicine OR exp Telecommunications OR exp Computer-Assisted Instruction OR exp Public Health Informatics OR exp user-computer interface OR exp Telephone OR exp Electronic Mail |
| 9 | (computer communication network OR computer-network OR computer-based OR computer-generated environment OR cyber OR cyberspace OR data highway OR electronic OR electronic mail OR email OR e-mail OR information highway OR information super  highway OR information superhighway OR internet OR internet-based OR net OR online OR virtual OR web OR web-based OR webbased OR web page OR webpage OR web site OR website OR world wide web OR www OR chat group OR chatgroup OR chat room OR chatroom OR mobile phon* OR smartphon* OR cellular phon* OR text messag* OR "short message service" OR texting computer* OR microcomputer* OR laptop* OR ipad* OR internet* OR local area network* OR lan OR world wide web OR www OR web* OR worldwide web OR website*).ti,ab. |
| 10 | (health information technolog* OR medical information science* OR ((medical OR clinical OR health) adj2 informatics) OR telemedicine OR tele medicine OR tele health* OR telehealth* OR phone* OR telephone* OR electronic mail* OR e-mail* OR email* OR e-health OR electronic health OR ehealth OR online OR on-line OR chat room* OR chatroom* OR blog* OR web log* OR weblog* OR bulletin board* OR bulletinboard* OR messageboard* OR message board* OR twitter OR tweet* OR facebook OR yahoo OR skype OR youtube OR itunes OR podcast* OR iphone* OR ((app OR application) adj10 (internet OR online OR web*))).ti,ab. |
| 11 | 8 OR 9 OR 10 |
| 12 | 7 AND 11 |
| 13 | 1 AND 6 AND 12 |
| 14 | limit 13 to English language |
| 15 | limit 14 to humans |
